# Immune System as a Sensory System

**Published:** 2010-09

**Authors:** Igor M. Dozmorov, D. Dresser

**Affiliations:** 1 *Department of Arthritis and Immunology, Oklahoma Medical Research Foundation, Oklahoma City, OK, USA;*; 2 *The Ashworth Laboratory, School of Biological Sciences, University of Edinburgh, West Mains Road, Edinburgh, EH9 3JT, UK*

**Keywords:** lateral inhibition, receptor specificity, uncertainty, affinity

## Abstract

As suggested by the well-known gestalt concept the immune system can be regarded as an integrated complex system, the functioning of which cannot be fully characterized by the behavior of its constituent elements. Similar approaches to the immune system in particular and sensory systems in general allows one to discern similarities and differences in the process of distinguishing informative patterns in an otherwise random background, thus initiating an appropriate and adequate response. This may lead to a new interpretation of difficulties in the comprehension of some immunological phenomena.

## INTRODUCTION

To perform its functions, the immune system must ensure continuous reception and processing of information about the antigenic state of the organism. This perception must allow an evaluation of how serious is any divergence from the norm (the notion of “norm” of course varies during the development of the organism and between different organisms). The immune system initiates effector functions aimed to form an adequate defensive response. In the context of its “goals”, the immune system can be considered as a sensory organ receiving and processing specific information. Although this concept has been formulated by others (3, 10, 22, 41, 44), it has not however contributed to a new understanding of the laws regulating the functions of the immune system functions, nor has the immune system been put on a par with other sensory systems More over the immune system has sometimes been declared an isolated and peculiar system and thereby been denied any analogy with neuronal networks altogether (51, 64). In contrast to this firmly rooted view we want to demonstrate some results of establishing common laws that regulate the functioning of the immune system as well as other sensory systems of the organism.

## SENSORY SYSTEM STRUCTURE AND FUNCTIONING

Despite all the differences between sensory systems, typically they perform their functions using a single set of principal mechanisms (35, 57). Sensory systems allow for the continuous reception, processing and analysis of information sent by receptors after physical and chemical stimulation from the outer and inner environment of the organism. Evaluation of the full picture shows that sensory systems have an adequate response to the stimulus on the basis of the previous experience stored in their memory matrices. Generally, all sensory systems are characterized by pairs of opposite properties, such as high sensitivity and resistance to interference; sensitivity to a wide range of signals and fine discrimination between a variety of stimuli. These properties are essentially due to a specific structural organization of sensory system elements, which can be divided into the following levels:

- Sensory receptors;
- Information pathways;
- Neuronal networks to perceive and process sensory information.

Receptors transform the energy of the stimuli they have received into a sequence of frequency-modulated impulses suitable for their further transmission by the neuronal pathways. Such information is reported to display high resistance to interference (57). The relevant point is that the number of perceived characteristics of various stimuli exceeds considerably the actual number of specific receptors. This further implies that an accurate analysis of separate features of sensory stimuli cannot be based on the data from one receptor element. This also means that an increase in specificity could be accomplished through a cooperative activity of many elements of all sensory system levels.

Primary processing of information can take place already at the level of sensory receptors. Here, each sensory cell integrates the totality of received stimuli into a successive chain of events and displays an ability to adapt itself to the perceived impact (57).

An opportunity for finer processing begins during the passage of signals by transmission of of information through pathways such as those represented by chains of neurons connected into a central nervous system The discriminatory relationships within this network allow transmitted information, from one receptor element, to be diverted to different neuronal pathways. Thus, a great number of elements can become involved in information processing. Converging in one neurone, these associations allow it to receive and integrate information from several sources. All receptor cells that have an impact on this neuron constitute its receptive field. Accordingly, any growth in the size of the receptive field raises receptor sensitivity to stimuli, although its localization is still not clear.

The existence of diverging and converging structural links can per se trigger a strong spread of the excited state. This implies that a weak sensory stimulus might be enough to trigger a chain reaction throughout the central nervous system. However it does not usually happen due to lateral inhibition.The restriction of the spread of excitation, the presence of receptive fields around central neurons, and the organization of information transfer, are due to lateral inhibition, i.e. inhibitory interaction between neurons of one level (21). The intensity of lateral inhibition is directly proportional to the excitation degree of the sensory element and inversely proportional to the excitation distance between receptor elements, or neurons, present at various levels of the sensory system. Here, "distance" is meant the value used to describe the degree of comprehended information (for example geometric distance for visual images, frequency for audio images etc.). Projecting a visual image onto the receptive field, the receptor cells bordering on the line between light and dark undergo the following changes. When activated by illumination they suppress the activity of their neighbors. As a result, the shaded receptors next to the borderline become even more retarded than those amid unstimulated receptor cells. Accordingly, receptors within the illuminated area of the receptive field adjacent to the shaded receptors appear to be more activated than those in the heart of the illuminated area since the former at least partially avoid suppression by their neighbors. Thus the sensory system, by using lateral inhibition, positively distorts images through their contrasts, i.e. accentuating their most dominant features and making image recognition and comparison significantly easier. Signal processing via lateral inhibition takes place throughout the entire transmission path and ends with information analysis in central organs (the cortex in higher animals).

Not only does the system respond to specific stimuli but also to the stimuli which are currently insignificant background and which must be inhibited. To form an adequate reaction toward novel environment and novel factors the organism has to start a special analysis of stimuli, compare them with the past experience and find appropriate behavioral responses. In other words, the organism has to be able to adjust itself for acceptance of new stimuli. When new “filters” are in place, the formation of a specific reaction is relatively straightforward and semi-automatic with the repetition of learned tasks. Fine analysis is a property of the cooperative work of a great number of nervous-system elements associated into a network. According to the widely popular concept of the probability organization of the brain nervous networks, the neuronal networks are functionally indeterminate dynamic structures which are assembled for a specific purpose and decompose as soon as this purpose is achieved. After disengagement, these very neurons can re-organize in new functional networks. Thus, the virtually unrestricted variety of brain activities is based on a relatively small number of neural cell types.

## LAWS OF IMMUNE SYSTEM FUNCTIONING THROUGH THE PERSPECTIVE OF THE SENSORY SYSTEM THEORY

Cunningham was among the first to transfer the gestalt concept from general biology to immunology (18). According to gestalt concept, the work of a complex system could not be fully understood by the work of its isolated parts. This suggests that following a classical way of decomposing a system into its simple constituents (reductionism) may lead to a gap in our understanding of the global system functioning. “Complex systems are complex precisely because they resist reduction; their properties of interest are emergent properties” (34).

The idea that the immune system as a whole has more attributes than the simple sum of its parts has won many supporters and has being increasingly substantiated by new evidence. The behavior of immune system elements is characterized by coherence implying that each element acts as if it possesses a totality of information available to all its elements and as if its sense of appropriateness of the adopted behavior originates not from one element alone but by the system overall (11, 12, 15, 48). It is unlikely that such behavior would be predictable in a system that lacks close interaction between its elements. The immune system contributes to such interaction by forming a non-specific humoral (cytokines) background and generating specific interconnections. Quintessential, however, has become the concept that immune elements are associated into network. According to modern concepts based on connectionism, "the network itself decides how to tune its component elements in mutual relationships that give the entire system a capacity (recognition, memory, etc.) which is not available to the components in isolation" (64).

We hope to show here that the immune system, as a sensory system, is able to solve similar problems of perception, processing of information and appropriate response by using principally the same set of behaviour patterns (Table 1):

- The perception of new information which variety exceed considerably the actual number of receptor type (sensitivity at the expense of selectivity);
- Contrasting of the antigenic image in course of immune response maturation (lateral inhibition);
- The use of receptors differing in their adapting abilities to incoming stimuli (complementarity principle).

**Table 1:** Common solutions of the similar problems of perception and processing of information in sensory systems and in the immune system

	Reception	Prevention of signal dissemination	Information processing	Image contrast	Receptor adaptation

Sensory systems	High sensitivity, Low specificity	Lateral inhibition - inhibitory interaction between adjacent sensory neurons	Transmits sensory information collected by receptors to the CNS	Lateral inhibition throughout the entire transmission path, ending with information analysis in CNS sensory centers	Tonic receptors - slowly acting, no adaptation.
					Stimulus = the presence of signal. Firing until the stimulus is here.
					Phasic receptors -rapidly adapting;
					Stimulus = the change of the signal
					Stop firing when stimulus is constant.
Immune system	The number of antigen specificities which the immune system can perceive and detect may exceed the existing receptor-cell variety at any given moment	Elements of lateral inhibition in cell-cell communications.	Coherence through interaction by forming a non-specific humoral background and generating specific interconnections	Immune response maturation increase of cell specificity.	Tonic: specificity-high; sensitivity-low
		Antigen competition.		Bimodal distribution of antibody affinity	Stimulus = the presence of the foreign antigen.
		*Notch* signaling. Prevention of immune response to self antigens by generating of regulatory T cells			Phasic: specificity-low; sensitivity-high
					Stimulus:
					= temporal variation;
					= spatial variation (appearance of any antigen in non adequate environment - spatial heterogeneity).

## THE RECEPTIVE FIELDS OF THE IMMUNE SYSTEM

The structural organization of the immune system is characterized by very similar elements to those which are typical for other sensory systems. Iimmunocompetent cells have receptors able to differentially sense antigenic information. The immune system also has the mechanisms for encoding and transportation of information,which take the form of antigen processing followed by analysis of information at the level of stable forms of peptide fragments in association with molecules of the main histocompatibility complex (MHC) located on the membranes of the antigen-presenting cells (53).

Overlapping specificities are characteristic of sensory systems in general and the immune system in particular. The number of antigenic specificities which the immune system can eventually perceive and detect is in practical terms essentially limitless due to the generation of the diversity mechanism (53). As a rule, high sensitivity and comprehensive information perception at the early stages are attainable at the expense of selectivity. Some lymphocytes – present in vivo in an activated form may take on the role of receptor cells during the primary immune response. In the case of the humoral immune response the receptor cells will be B1a-lymphocytes that are initially responsible for forming the basic specific IgM background in a serum and for natural antibody secretion (7, 23, 32). Such antibodies expressed on the cell surface early in a response display polyreactivity and low affinity to auto- and exogenous antigens in contrast to generally high-affinity antibodies (mainly IgG) which appear during a mature phase antigen-evoked response. The increase of affinity of these receptors may involve a competitive selection, mediated by decreasing free antigen (53).

Turning to T- lymphocytes, one may note the same dichotomy, where the role of primary receptor cells may be assigned to naturally activated blast cells which comprise about 20% of the total number of T cells, and which in the primary response perform a regulatory function, while their counterparts - small resting T lymphocytes - act as effectors (13, 61).

During a humoral immune response there is an increase in non-specific background (rheumatoid factor) which parallels a similar increase in non-specificity seen in other sensory systems. Such an increase in non-specificity may derive from a state of excitation spreading from cell to cell. It has been hypothesized that the increased glucocorticoid blood levels, observed at or near the time of peak immune response, might prevent the non-specific spread of activation and contribute to the formation of a specific immune response (8, 9, 19, 59). There are also other potential mechanisms, some of them similar to lateral inhibition, which could also contribute to the prevention of non-specific lymphocyte activation.

## CONTRASTING OF THE ANTIGENIC IMAGE IN COURSE OF IMMUNE RESPONSE MATURATION

The initial phase of immune response as a perception phase of any sensory process should be and is of very low specificity. It can be expected that in course of immune response specificity will be increased as a result of selection processes. Under conditions of decreasing concentrations of antigen the degree of affinity selection takes place such that only lymphocytes that can respond to low concentrations of antigen give rise to memory cells. Indeed in the course of the antigen-specific immune response, the affinity of serum antibodies grows with time, a phenomenon which is known as immune response maturation (52). Now there are obtained evidences that similar process of response maturation take place also in case of T cell response. The development of T cell immune response is also includes selection and accompanied by reduction in the heterogeneity of TCR gene usage (68). The repertoire of these cells is less diverse and therefore less crossreactive than the primary one but it still does not explain the specificity of immune response. Increased affinity of immunocompetent cells for initiating antigen is not necessary associated with increase specificity of these cells. Affinity or the strength of binding between receptor and ligand is not a synonym of specificity or ability to discriminate antigens of different proximity. Considerable crossreactivity such that one TCR can recognize a number of different peptides that do not necessary show strong sequence homology (16, 45) is demonstrated for T cells passed through several cycles of activation-rest selections.

However all immune response maturation theories, though based on the idea of the antigen-dependent positive selection may not explain satisfactorily the developmental mechanisms of the immune response maturation. No intelligible explanation has been given to the transformation of the original broad Gauss distribution of affinity produced antibodies (70) and of cells by their functional activity (32) to a bimodal distribution at the final stage of the response. Positive selection alone is apparently unable to explain the absence of many medium affinity types in the final pattern of antibody affinities.

Drawing a parallel between memory formation and information processing in sensory systems, enables an interpretation of immune response maturation as the processing of information through sensitivity to contrasting characteristics. Probably the participation of lateral inhibition in information processing leads to a fierce competition among leading options thus facilitating selection of the best receptor/ligand fit. In addition, lateral inhibition may provide a conceptual basis for an explanation for the preservation of clones either expressing low-affinity receptors or completely unable to bind to the initiating antigen. In other words those receptor cells which escape selection because their niches do not overlap (1, 6).

To trigger a highly specific effector phase of an immune response one might expect that there should be a cooperative mechanism analogous to the lateral inhibition in other sensory systems. The concept of lateral inhibition is not a pre-requisite of neuronal interactions and has been adapted, for example to describe processes in the development of cell types and biological pattern formation (5, 64)0. It is a type of cell–cell interaction whereby a cell that enters a particular differentiation pathway inhibits its immediate neighbours from entering the same pathway. The transmembrane proteins Notch and Delta (or their homologues) have been identified as mediators of the interaction (36-38).

Lymphocytes responding to antigen stimulation should have an opportunity to interact with each other directly or through regulatory cells. This interaction should be in favour of most activated cells bearing receptors high affinity for antigen. Activity of cells with lower affinity receptors for a given antigen should be inhibited. It is possible that factors such as Notch and Delta play a role in lateral inhibition among immune cells (14, 20, 38).

What known facts confirm the participation of the lateral inhibition mechanism in the antigenic image contrasting. An inhibitory interaction between T lymphocytes responding to antigenic stimulation was demonstrated earlier (69). This phenomenon was especially clearly established when some of them were tolerated and did not produce their own effector activity. Anergic T cells acted as active suppressors of T cell responses when had chance for specific recognition of antigen presented on the same APC (33, 50, 63, 65, 67). It suggests that there is mechanism for mutual inhibitory influence of antigen activated cells resembling lateral inhibition mechanisms of sensory systems (example of the role of lateral inhibition in cell fate was presented in (14, 20, 39)). This mechanism can promote competitive victory of cells with highest complementarily of their receptors for given antigen structure. The same mechanism is able to improve specificity of “winners”, because their activity to proximal structures (alterations) will be inhibited by “losers”, who have receptors with higher complementary to these alterations. This sharpening can be especially important for avoiding of autoreactivity from mature effectors. Presence of anergic self-reactive effectors appeared to be necessary for prevention of autoreactivity from mature effectors (there are data that purging from organism of autoreactive clones makes it vulnerable to autoimmune pathologies (17, 40, 71).

In case of humoral immune response the evidence for contrasting through lateral inhibition may be obtained in the next fact of transformation of the original Gauss broad-disperse distribution of produced antibodies according to their affinities (37, 70) and of cells by their functional activity (29) to a bimodal distribution at the final stage of the response. This bimodality resembles the profile of the preception on the border between bright image and dark periphery appeared as a result of inhibitory interaction between neighboring receptors. There is no information about negative interaction between B lymphocytes of close specificity to antigen, however one can hypothesize that regulator T-cells [Tregs] are part of the lateral inhibition mechanism in this case (47). To take part in selection, T regs should be capable of distinguishing immunoglobulin-like receptors on the surface of both T and B cells differentiated on the basis of affinity related idiotypic epitopes (62). Compatible with our supposition, each B-cell clone recruited in the response may have a corresponding clone of “own” helper T cells (Tregs) whose activity may overlap with different but related clones of B-cells, which is possibly an example of lateral inhibition. According the conception of lateral inhibition it appears that “loser clones” or self-reactivity clones that are not annihilated during maturation and competitive selection could be the part of this mechanism. Although their effector function is regulatory suppressed, these clones, by their simple presence (via regulatory T lymphocytes), participate in the formation of a specific response. Unfortunately, there is few experimental data in support of this supposition.

Indirect evidence for lateral inhibition as a common process in any antigen specific response was obtained in limiting dilution analysis. In many experimental systems the dependence of the proportion of negative cultures against responder cell input has nonlinear zigzag shape (26, 43). In each of these cases, an initial seemingly linear dose-response relationship is followed by a distinct kink and a region of positive slope where increasing cell numbers lead to decreasing proportion of positive cultures. The curve at higher cell dose reverts to a second region of responsiveness. It was interpreted as evidence for competition of two different types of limiting cell types (24) one of which has a first order kinetics of activity and responsible for the initial linear decrease of the proportion of negative cultures. The second cell type has multi-hit effector kinetics but able to inhibit the first type cell activity in common cultures. This proposition about the mechanism of zigzag formation was realized in form of the mathematical model (24, 26), and was also confirmed experimentally (25, 27). However the same dependence could be raised from the mixture of responding cells of different TCR affinity (as in normal composition) if there was reciprocal inhibition between activated cells. In this case responding cells are able to demonstrate single hit kinetics when are single in cultures (initial linear part of LDA plot), but only cells with highest receptor affinities will produce positive cultures at higher input. The price for the victory – they will demonstrate multi-hit kinetics, when only summary activity of several such winners will produce positive cultures. It means that zigzag LDA plot may be an indirect evidence of the lateral inhibitory interactions in heterogenous responding population and the universality of zigzag (T helper functions, MLR responses, CTL responses, humoral responses ea, (24-27, 43)) only stresses the importance of recognition contrasting for mature phase of response.

## THE PRINCIPLE OF COMPLEMENTARITY IN THE IMMUNE SYSTEM (SELF-NON SELF DISCRIMINATION)

**Figure 1:**
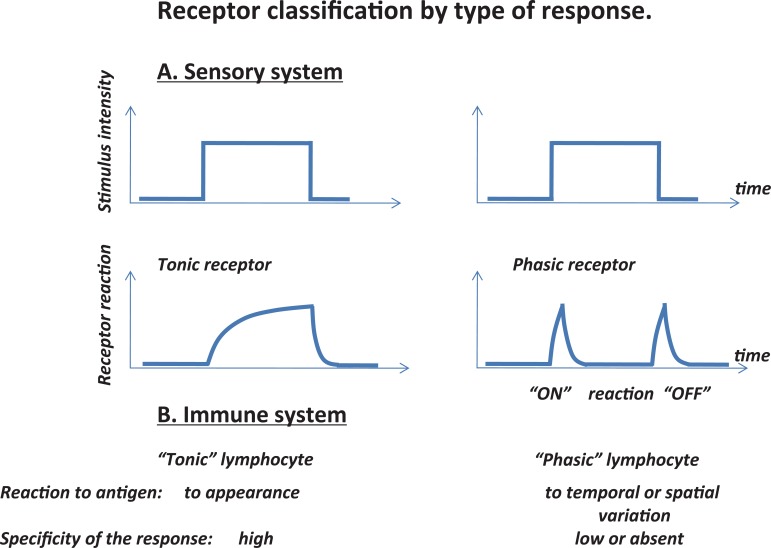
Two types of receptions differing by the rate of adaptation to the dynamical stimulus. A, Sensory system have two types of receptors differing by the rate of adaptation (9): tonic receptors that adapt slowly to a stimulus and continues to produce action potentials over the duration of the stimulus (left), and phasic receptors that adapt rapidly to a stimulus (right) and react to both the emergence of the signal (on- reaction) and to it cessation (off-reaction). The response diminishes quickly ant then stops. B, Immune system has different types of cells resembling in their reactions of tonic and phasic receptors of sensory systems. Some lymphocytes react preferably to the presence of foreign antigen (CD5- B lymphocytes, small resting T lymphocytes), whereas the others react to the change of antigenic contest in time (CD5+ B lymphocytes, naturally activated T-blast forms) or to the appearance of heterogeneity in presumingly homogenous tissue (NK killing of syngeneic targets in nonsyngeneic for targets environment (64, 65)).

It has been reported that sensory systems have two types of receptors (35): 1) phasic receptors rapidly adapting and reacting mostly to the switching on and off of stimuli and 2) slowly adapting tonic receptors. It would appear that they are able to sense two basic characteristics of signal, tonic receptors detecting the amplitude of the signal and phasic receptors detecting the rate of change of the signal. Hence, the necessity of two specialized structures for sensing these two additive parameters can be viewed as an expression of complementarity in the physiology of sensory systems, a principle more or less analogous to the Bohr’s principle of complementarity in quantum mechanics. This principle of quantum theory states that a physical object can have pairs of complementary or conjugate properties, but we can only perceive one at a time and are never able to make simultaneous predictions of conjugate variables, such as, for example position and momentum. Gabor (36) was the first who noted the intriguing fact that in psychophysics, as in quantum physics, one could accurately determine conjugate variables (time of the signal occurrence or its frequency) one at a time, but not both (36, 60). Thus a complementarity principle holds for psychophysics as well as for quantum physics (Fig. 1).

Although the relationships between the immune response and the amount of antigen have been thoroughly investigated (30, 31, 42) the dependence of this reaction upon the rate of alteration of the antigenic stimulus has until lately escaped specialist attention (4). The complementarity principle is now extended to the immune system. In humoral immunity analogies may be drawn between a membrane receptor such as CD5- and tonic receptors in a sensory system and between CD5+ and phasic receptors. In the T cell compartment, phasic receptor cells may arise from naturally activated splenic T-blast forms and act as regulators in a primary response, while their counterparts, small resting T lymphocytes may act as tonic effectors. Activity of CD5- cells directed towards the eventual elimination of foreign antigen slowly adapting as long as the antigenic signal lasts. The rapid reaction of CD5+ cells to disturbance caused by external or internal stimuli, is directed towards the quick neutralization of the stimulus. Neither specificity nor the degree of foreigness seem to play an important role in this reaction; only change in antigen concentration can achieve this. It therefore seems likely that cells can respond as well to change of both foreign to the body and endogenous or self antigens, thus being capable of operating as a first line of defence against foreign invasions or somatic mutations. Single mutated cell carries rather small quantity of new antigen, but the appearance of the unexpected antigenic structure within homogeneous pool of tissue cells is a strong alteration of homogeneity and therefore may be a significant stimulus for phasic perception (54-56). The mutated antigen should be not necessary foreign in general sense, but “foreigh” for given tissue environment. The phasic stuimulus with relatively low antigenic specificity/affinnity, may be of importance for the “adjuvanticity” response controlling entry to the tolerance or immunity pathways (28, 30) and a reinterpretation as a “dangerous” versus “friendly” signal as a key signal to control of responsiveness (46).

The immune system performs two fundamental functions: defense against invasion of foreign antigens and maintenance of stability of the internal antigenic range (53). This implies that an alteration, whether quantitative, qualitative or a disturbance of homogeneity of the antigenic milieu, could be the key characteristic which helps the immune system to discriminate between exogenous and endogenous antigens. This hypothesis is compatible with the formation of the immune response to self-antigens under an artificial situation caused by abrupt change in self-antigens. It is worth noting that receptor elements can react to the emergence of the signal (on-reaction) and its cessation (off-reaction) (Fig. 1). The formation of the immune response to self-antigens in animals after the removal of an own organ (partial hepatectomia or the removal of one own kidney) can serve as an illustration of the off-reactions (2, 49, 58). This evidence is compatible with the view that tolerance as well as immunity is dependent on the persistence of antigen.

## CONCLUSION

Consideration of the immune system as a sensory system helps in the establishment of a logical basis for connections between activation and function of receptor cells. This may help in a new interpretation of many obscure immunological phenomena. The existence of coherent functioning of several receptor elements may clarify the paradoxical ability of the immune system to recognize more specificities than are genomically accountable. The involvement of the lateral inhibition mechanisms during maturation of an immune response helps to explain the high specificity of the immunological memory and a degree of insurance against autoreactivity, in spite of the high degree of autoreactivity seen in a primary response. Finally, interpretation of immunocompetent cells in terms of phasic and tonic receptors enables one to establish the existence of two basic functions of the immune system: defense and self-maintenance. An interpretation based on an analogy with other systems suggests theoretical extrapolations allowing to look at the well-known facts from a new viewpoint leading closer to a comprehensive understanding of the immune system functions.
